# *Anti*-*Helicobacter pylori* and antiulcerogenic activity of *Aframomum pruinosum* seeds on indomethacin-induced gastric ulcer in rats

**DOI:** 10.1080/13880209.2017.1285326

**Published:** 2017-02-05

**Authors:** Laure Brigitte Kouitcheu Mabeku, Blandine Nanfack Nana, Bertrand Eyoum Bille, Roland Tchuenteu Tchuenguem, Eveline Nguepi

**Affiliations:** a Microbiology and Pharmacology Laboratory, Department of Biochemistry, Faculty of Science, University of Dschang, Dschang, Cameroon;; b Gastroenterology Department, Laquintinie Hospital of Douala, Douala, Cameroon

**Keywords:** Gastroduodenal diseases, non-steroidal anti-inflammatory drugs, herbal drugs

## Abstract

**Context:** Peptic ulcer is one of the most common diseases affecting mankind. Although there are many products used for its treatment, most of these products produce severe adverse reactions requiring the search for novel compounds. Some *Afromomum* species are used traditionally to cure acute gastritis.

**Objective:** To evaluate the antiulcer activity of the methanol extract of *Aframomum pruinosum* Gagnepain (Zingiberaceae) seeds against two major etiologic agents of peptic ulcer disease; *Helicobacter pylori* and non-steroidal anti-inflammatory drugs.

**Materials and methods:** The anti-*Helicobacter* activity of *A. pruinosum* was evaluated using the broth microdilution method. After oral administration of indomethacin (5 mg/kg) for 5 consecutive days, gastric ulcerated animals were divided into control group and five other groups: three groups that recieved respectively 125, 250 and 500 mg/kg of plant extract, the fourth group received Maalox (50 mg/kg) and the fifth group, Misoprostol (100 μg/kg), respectively, for 5 days. Ulcer areas, gastric mucus content and nitric oxide gastric levels of animals were assessed 24 h after this treatment.

**Results:***A. pruinosum* extract shows a moderate anti-*Helicobacter* activity with an MIC value of 128 μg/mL. *A. pruinosum* extract, like Misoprostol and Maalox, markedly reduces the % of ulcerated area from 8.15 ± 0.33 to 1.71 ± 0.44% (500 mg/kg). It also increased significantly mucus and NO gastric production with respective values of 4.44 ± 1.35 and 965.81 ± 106.74 μmol/g (500 mg/kg).

**Discussion and conclusion:** These findings suggest that *A. pruinosum* methanol extract possesses antiulcer properties as ascertained by the comparative decreases in ulcer areas, increase of mucus and NO gastric production.

## Introduction

Gastric ulcers are defined as a breach in the mucosa of the alimentary tract, which extends through the muscularis mucosa into the submucosa or deeper (Manonmani et al. 1995). The gastric mucosa is constantly exposed to harmful agents such as drugs, pepsin, gastric acid, bile acids, food ingredients (Toma et al. 2005). These causative factors have been associated with the pathogenesis of gastric ulcerations by means of pronounced gastric acidity, increased inﬂammatory markers and cell proliferation along with reduced gastric motility and gastric blood ﬂow (Pradeepkumar Singh et al. 2007).

Non-steroidal anti-inflammatory drugs (NSAIDs) such as indomethacin are known to cause gastric ulcers, especially when abused (Belaiche et al. 2002). The pathogenesis of NSAIDs-induced gastric ulceration includes the NSAID blocking the activities of the cyclooxygenase enzymes (COX-1 and COX-2), hence leading to reduced mucus and bicarbonate secretion, decreased mucosal blood flow, impaired platelet aggregation, alteration of microvascular structures leading to epithelia damage (Wallace et al. 2000).

*H. pylori* is a major etiologic agent of peptic ulcer disease, primary gastritis, gastric mucosa-associated lymphoid-tissue lymphoma, and gastric adenocarcinoma (Brown 2000). Eradication therapy of symptomatic *H. pylori* infection substantially reduces the recurrence of associated gastro-duodenal diseases. In general, triple therapy regimens usually entail two of the following antimicrobial agents: metronidazole, amoxicillin, tetracycline, or clarithromycin in combination with a proton pump inhibitor or bismuth (Hoffman & David 2001). However, *H. pylori* is still a difficult infection to eradicate as failure rate remains at 10–40% (Lai et al. 2006).

Although there are many products used for the treatment of gastric ulcers, most of these drugs produce several adverse reactions (Ariyphisi et al. 1986). In addition, the growing problem of antibiotic resistance by *H. pylori* drastically increased the frequency of therapeutic failure (Tanih et al. 2010) and demands the search for novel compounds from plant based sources.

In Cameroon, 20 species of the genus *Aframomum* (Zingiberaceae) are found and are widely used for medicinal, ethno-dietary, cultural and spiritual purposes (Amvam Zollo et al. 2005). Among them, *Aframomum pruinosum* Gagnepain is popularly used to cure several afflictions such as women sterility and schizophrenia; they might also have antisympathetic properties and tranquilizing effects (Tane et al. 2005); there is a general agreement on their properties to bring strength and peace in families with twins (Nguikwie et al. 2013). Chemical investigations on essential oils from *Aframomum* species revealed that *A. pruinosum* seed oils were rich in (*E*)-nerolidol, while the leaf essential oil was dominated by sesquiterpenes (Amvam Zollo et al. 2005). Antibacterial activities of the rhizome oils of *A. pruinosum* against *Escherichia coli* are also documented (Nguikwie et al. 2013). As far as the literature survey could ascertain, antiulcerogenic activity of *A. pruinosum* has never been reported before, despite the fact that other members of *Afromomum* genus such as *Afromomun citratum* Pereira and *Afromomum melegueta* Koechlin are used traditionally to cure acute gastritis (Adjanohoun et al. 1996). The present study has, therefore, been conducted to evaluate the anti-*H. pylori* and antiulcerogenic effect of *A. pruinosum* methanol extract on NSAID induced gastric lesions in rats.

## Materials and methods

### Plant material

The fruits of *A. pruinosum* were collected in January 2015 in Fontem, West Region of Cameroon. Identification of the plant was done by M. Victor Nana of the National Herbarium (Yaounde-Cameroon) where the voucher specimen was available under the reference number no. 10880 SRF/Cam (Sociétés des Réserves Forestières du Cameroun). The plant materials were then air-dried at room temperature and the seeds were removed and ground into a fine powder.

### Preparation of plant extract

The powdered plant material was weighed (300 g) and soaked in 1 L of methanol (MeOH) or ethyl acetate (EA) for 72 h at room temperature. The filtrate obtained through Whatman filter paper no. 1 was concentrated under reduced pressure in a vacuum to obtain the crude extract. All crude extracts were kept at 4 °C until further use.

### Preliminary phytochemical analysis

This was done according to the method proposed by Parekh and Chanda (2008) to determine the presence of flavonoids (alkaline reagent, Shinoda), phenolics (lead acetate, alkaline reagent test), triterpenes and steroids (Liberman-Burchard test), saponins (foam test), tannins (gelatine), anthraquinones (ether-chloroform 1:1 v/v, NaOH 10% w/v) and anthocyanins (1% HCl, heating). The results were qualitatively expressed as positive (+) or negative (−).

### Anti-*H. pylori* assays

#### Chemicals for antimicrobial assay

Doxycycline (200 mg, Combitic Global Caplet Pvt, India), erythromycin (erythromycine stearate 500 mg, Cipla Ltd, India), amoxicillin (amoxicillin trihydrate 500 mg, Maxheal Pharmaceutical Ltd, India) and ciprofloxacin (ZOFLOX, ciprofloxacine 750 mg, Odypharm) used as reference antibiotics were purchased from a local pharmacy. *p*-Iodonitrotetrazolium chloride (INT, Sigma-Aldrich) was used as a microbial growth indicator (Mativandlela et al. 2006). Columbia agar supplemented with 5% (v/v) lacked horse blood (SR0048 Oxoid, Basingstoke, England) and 1% (v/v) Vitox was used for the activation of the tested clinical strains while Brian Heart Infusion (BHI) broth supplemented with 5% horse serum was used for antibacterial assays. Campy*Gen* gas park (CN0024A Oxoid, Basingstoke, England) was used to generate microaerophilic conditions of culture.

#### Microbial strains

A total of six strains of *H. pylori* were freshly isolated from gastric biopsies of patients with gastric-related morbidities undergoing endoscopy at Laquintinie Hospital in Douala-Cameroon. The study was approved by local ethical committee of Laquintinie Hospital (Approval no. 425/AR/MINSANTE/HLD/SCM/CR) and the specimens were only collected from patients who had given consent. The isolates were identified by Gram staining and enzymatic activity (catalase, oxidase, urease) (Miendje Deyi et al. 2011). Pure cultures were suspended in Eppendorf tubes containing 1 mL of Brian Heart Infusion broth supplemented with 5% horse serum and 20% glycerol and stored at −80 °C until used.

#### INT colorimetric assay for MIC and MBC determinations

The plant extracts were further used for the determination of MICs by INT (*p*-iodonitrotetrazolium chloride) broth microdilution method (Mativandlela et al. 2006) (Table 1). The micro dilution test was performed in 96-well plates. Two-fold dilutions of the extract were prepared in the test wells in Brian Heart Infusion broth supplemented with 5% horse serum (BHI-serum). The final extract concentrations ranged from 2 to 1024 μg/mL. Inoculums (100 μL) at McFarlands turbidity standard 3, from a 48 h colony in Columbia agar supplemented with 5% (v/v) lacked horse blood and 1% (v/v) Vitox was then added to 100 μL of extract-containing culture medium. Control wells were prepared with culture medium and bacterial suspension, and broth only. Ciprofloxacin, doxycycline, erythromycin and amoxicillin at concentration ranges of 2–128 μg/mL were used as positive control. The plates were covered with a sterile plate sealer and then agitated with a shaker to mix the contents of the wells and incubated for 3 days at 37 °C under microaerophilic conditions. After incubation, 40 μL of 0.2 mg/mL INT was added to each well, and incubated at 37 °C for an additional period of 30 min. Viable bacteria reduced the yellow dye to pink. MIC was defined as the sample concentration that prevented the colour change of the medium and exhibited complete inhibition of microbial growth. The MBC was determined by adding 50 μL aliquots of the preparations which did not show any growth after incubation during MIC assays, to 150 μL of adequate broth. These preparations were incubated at 37 °C for 72 h under microaerophillic conditions. The MBC was regarded as the lowest concentration of extract, which did not produce a color change after addition of INT as mentioned above. All the experiments were carried out in triplicate.

**Table 1.** MIC/MBC of ethyl acetate and methanol crude extracts of *A. pruinosum* and antibiotics (μg/mL).

	Microorganisms					
Plant-extract/antibiotics	*H. pylori* α1	*H. pylori* α2	*H. pylori* α3	*H. pylori* α4	*H. pylori* α5	*H. pylori* α6
*A. pruinosum*(MeOH)	128/256	128/512	256/1024	512/512	512/1024	1024
*A. pruinosum*(AE)	256/1024	256/512	512/1024	1024/>1024	>1024	1024
Ciprofloxacin	8/8	8/8	32/32	16/32	16/16	4/16
Doxycycline	8/8	16/32	32/32	32/32	32/64	32/32
Erythromycin	32/32	16/16	32/32	128/128	>128	>128
Amoxicillin	8/16	16/16	16/16	4/16	>128	>128

All the values given in the table are means of triplicates determinations. EA: ethyl acetate; MeOH: methanol.

### Gastric antiulcer assays

#### Drugs

The following reference antiulcer drugs were used: Aluminum hydroxide (Maalox^®^, Sanofi Aventis, Paris, France) and Misoprostol (Cytotec^®^, Pfizer, Freiburg, Germany). Indomethacin (Saint Louis, MO) was used as ulcerogenic agent.

#### Experimental animals

Sprague–Dawley Wistar albino healthy adult male rats (150–200 g) obtained from the Animal House, University of Dschang-Cameroon, were used for the study. The rats were given food and water *ad libitum*. These animals were used for the non-steroidal anti-inflammatory drug-induced gastric ulcer assessment. Animal housing and the bioassay was conducted in accordance with the internationally accepted principle guidelines of the European Union on Animal Care (CEE Council 86/609). The Cameroon National Ethical Committee approved the protocols of the study (Ref no. FW-IRB 00001954).

#### Ulcer induction

Forty-two rats were allowed to acclimatize for one week before initiation of experiment. The rats were randomly divided into seven groups of six animals. After 24 h of fasting, six of the seven animal groups received indomethacin (5 mg/kg) for five consecutive days in order to induce gastric ulcer and one group was kept as normal control. Twenty-four hours after the last treatment with indomethacin, ulcer control group was given 1 mL of vehicle (1% aqueous solution of Tween-80), reference groups received oral doses of 50 mg/kg Maalox or 100 μg/kg Misoprostol as positive control and the remaining three groups received 125, 250 and 500 mg/kg of *A. pruinosum* methanol extract, respectively. All the treatments were administered orally for five days. The rats were euthanized under an overdose of ether anaesthesia 24 h after the last day of drug treatment. The abdominal cavity of each animal was opened and organs such as stomach, liver and kidneys were quickly removed, cleaned with ice-cold saline and weighed. The stomachs were then opened along the greater curvature and ulcer scoring was done. The fundic part of the stomach was also used to estimate NO production.

#### Relative organ weight

The relative weight (ROW) of stomach, liver and kidneys of each animal was calculated as follows. ROW = [Absolute organ weight (g)/Body weight of rat on day of sacrifice (g)] x 100.

#### Measurement of mucus production

Gastric mucus production was measured in the rats that were subjected to indomethacin-induced gastric lesions. The gastric mucosa of each rat was obtained by gently scraping the mucosa with a glass slide and the collected mucus was weighed by using a precision electronic balance (Wasman et al. 2010; Ketuly et al. 2011).

#### Preparation of gastric homogenate samples

To prepare the tissue homogenates, the fundic part of the stomach was thawed and homogenized 20 times (w/v) by homogenizer in ice-cold carbonate 0.4M buffer (PH 10.2). The homogenates were centrifuged at 2000 rpm for 15 min and the supernatant was then used for determination of NO levels.

#### Estimation of nitrogen oxide (NO) level

Nitric oxide content was quantified by measuring nitrite/nitrate concentration using the Griess assay (Hiruma-Lima et al. 2006). In brief, gastric homogenates were deproteinated with absolute ethanol for 48 h at 4 °C, then centrifuged at 12,000*g* for 15 min at 4 °C. To an aliquot of the supernatant, vanadium trichloride 0.8% (w/v) in 1 M HCl was added for the reduction of nitrate to nitrite, followed by the rapid addition of Griess reagent (Sigma) and the absorbance at 546 nm was measured. The results were expressed in μmol/g tissue. Sodium nitrite was used as standard. All the tests were carried out in triplicate.

#### Gross gastric lesions evaluation

For the measurement of the gross gastric mucosal lesions, freshly excised stomach was laid flat and gastric mucosa of each rat was thus examined for damage. Ulcers of the gastric mucosa appeared as elongated bands of haemorrhagic lesions parallel to the long axis of the stomach. The mucosal lesions were then photographed and scored. To score, the length and width of the ulcer (mm) were measured with a ruler under magnifying glass and the ulcerated area of each ulcer band was calculated. The number and severity of erosions were scored using the scoring method as follows (Tan et al. 1996): 0 = No lesion, 0.5 = Haemorrhage, 1 = ulceration area ≤0.5 mm^2^, 2 = ulceration area between 0.5 and 2.5 mm^2^, 3 = ulceration area between 2.5 and 5 mm^2^, 4 = ulceration area between 5 and 10 mm^2^ and 5 = ulceration area >10 mm^2^. The results were expressed as ulcer index (UI), total ulcer area (UA), percentage of ulcerated area (UA%) and inhibition percentage (I%).

Ulcer index was determined using the following formula (Hoogerwerf & Pasricha 2001), (UI) = (UN + US + UP) × 10, where UI = ulcer index, UN = average number of ulcers per animal, US = average of severity score and UP = percentage of animals with ulcer.

The sum of the areas of all lesions for each stomach was applied in the calculation of the total ulcer area (UA).

The percentage of ulcerated area (UA%) was determined using the following formula (Tan et al. 1996): total ulcer area (UA)/total gastric area ×100, with the stomach considered as a circle with area = πd^2^/4.

The percentage protection or inhibition percentage was calculated according to the recommendation of Wasman et al. (2010):

Inhibition percentage (I%) = (UI control − UI treated group)/UI control ×100.

### Data analysis

All values were expressed as mean ± standard error. Statistical analyses were carried out using the software GraphPad Prism 5.01 (La Jolla, CA). The significance of the differences observed between the doses was achieved by analysis of variances (ANOVA) of the multiple tests of comparison of Tukey–Kramer. Differences between concentrations were considered statistically significant when *p* < 0.05.

## Results

### Anti-*H. pylori* activity of A. pruinosum extracts and antibiotics

*A. pruinosum* extracts showed different anti-*Helicobacter* activity each with MIC values ranging from 128 to >1024 μg/mL. The best activity was recorded with *A. pruinosum* methanol extract, with MIC values ranging from 128 to 512 μg/mL against 5/6 (83.33%) of the tested strains. MIC values from 256 to 512 μg/mL were also recorded with *A. pruinosum* (AE) extract against 3/6 (50%) of the bacteria tested. The lowest MIC value (128 μg/mL) was recorded with the methanol extract from *A. pruinosum* against *H. pylori-α1* and *H. pylori-α2.*

### Phytochemical screening

The results of the preliminary phytochemical screening of the methanol extract of *A. pruinosum* revealed the presence of phenols, tannins, triterpenes and anthraquinones (Table 2).

**Table 2.** Phytochemical screening of methanol extract of *Aframomum pruinosum*.

Compounds	Phenolics	Flavonoids	Steroid	Saponins	Triterpenes	Tanins	Anthraq	Anthoc
*A. pruinosum*	+	−	−	−	+	+	+	−

+: present; −: absent; Anthraq: Anthraquinones; Anthoc: Anthocyanins.

### Gastric antiulcer activity of A. pruinosum extract

#### Relative organ weight

There were no significant changes in the relative weights of the liver and kidneys of the treated rats in relation to control group. However, indomethacin administration produced a significant increase in the relative stomach weight of control animal group as compared to the normal group. Treatment with *A. pruinosum* methanol extract and reference drugs significantly reduced the gastric weight in animals with indomethacin-induced ulcers. Almost, all the treatments were able to reduce stomach weigh of gastric induced animal near to the normal group (Table 3).

**Table 3.** Effect of *A. pruinosum* methanol extract on the relative organ weights of indomethacin induced gastric ulcer rats.

Treatment	Stomach (10^−2^)	Liver (10^−2^)	Kidneys (10^−2^)
Normal group	0.80 ± 0.05**	4.24 ± 0.43	0.81 ± 0.03
Negative control group	1.12 ± 0.10	4.68 ± 0.79	1.00 ± 0.12
Maalox (50 mg/kg)	0.86 ± 0.15*	3.86 ± 0.41	0.93 ± 0.09
Misoprostol (100μg/kg)	0.90 ± 0.11*	4.15 ± 0.25	0.90 ± 0.07
*A. pruinosum* (125 mg/kg)	0.86 ± 0.14*	3.92 ± 0.63	0.88 ± 0.20
*A. pruinosum* (250 mg/kg)	0.88 ± 0.11*	4.34 ± 0.49	0.89 ± 0.06
*A. pruinosum* (500 mg/kg)	0.86 ± 0.10*	3.87 ± 0.20	0.93 ± 0.09

Each data column represents the mean ± SD (*n* = 6). Significantly different from the control group at *p < 0.05, **p < 0.01.

#### Gastric wall mucosal and nitric oxide evaluation

Administration of indomethacin caused a significant decrease in the mucus content of the gastric wall in the untreated animals (negative control group) compared to normal group (Table 4). Treatment with the methanol extract of *A. pruinosum* resulted in a concentration-dependent and significant increase of stomach mucus content of treated gastric ulcerated animals compared to untreated gastric ulcerated animals (control group) (Table 4). The effect of the extract at a lower dose (125 mg/kg) was similar to that of maloox (50 mg/kg) and misoprostol (100 μg/kg) as reference drugs used.

**Table 4.** Effect of *A. pruinosum* on gastric mucus content and NO level of indomethacin-induced ulcer rats.

Treatment	Gastric NO level(μmol/g tissue)	Relative mucusweight (10^−2^)
Normal group	942.83 ± 177.38*	1.02 ± 0.161*
Negative control group	623.72 ± 119.82	0.623 ± 0.083
Maalox (50 mg/kg)	877.70 ± 79.83*	2.14 ± 0.65**
Misoprostol (100μg/kg)	793.73 ± 102.54	2.73 ± 0.28**
*A. pruinosum* (125 mg/kg)	885.69 ± 73.47*	2.25 ± 1.03**
*A. pruinosum* (250 mg/kg)	973.75 ± 99.74*	3.54 ± 1.74***
*A. pruinosum* (500 mg/kg)	965.81 ± 106.74*	4.44 ± 1.35***

The results are mean ± SD for 6 animals/group. Significantly different from the control group at *p < 0.05, **p < 0.01 ();***p < 0.001.

As on mucus content, indomethacin-induced ulcer was also associated with a decreased level of NO in rat’s gastric tissue. We found that treatment with *A. pruinosum* methanol extract and reference drugs significantly replenished the depleted gastric NO level in such a way that its level was similar to that of a normal animal at highest plant-extract doses (250-500 mg/kg) (Table 4).

#### Gross appearance of the gastric mucosa in rats

**Figure 1.**
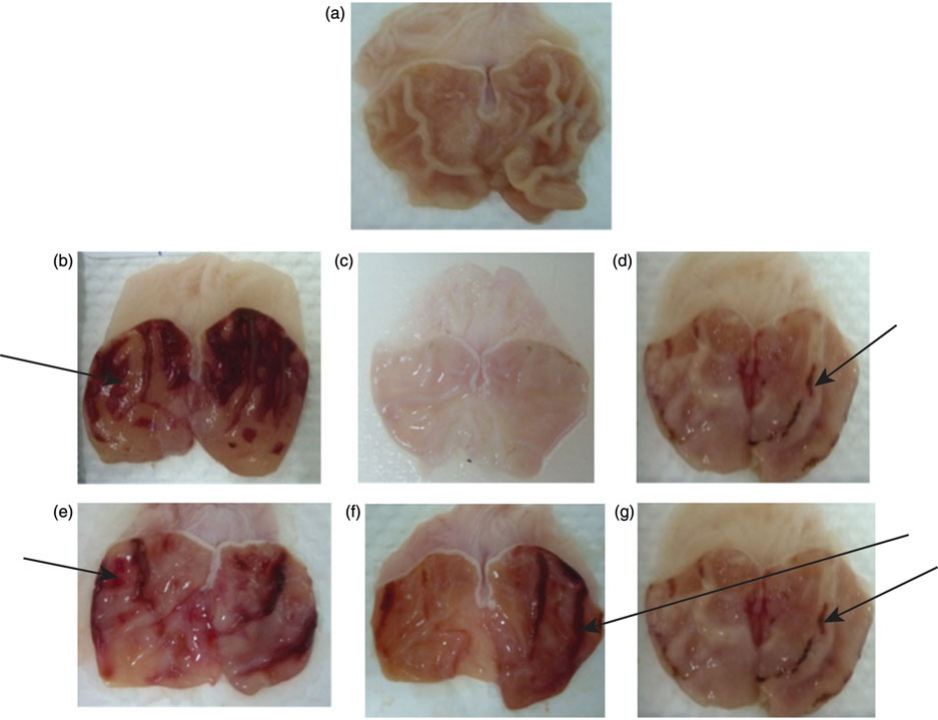
Gross appearance of the gastric mucosa in rats. Normal animals (a): treated with 1ml of vehicle (1% aqueous solution of Tween-80); ulcer control or negative control group (b): severe injuries are seen in the gastric mucosa (arrow), indomethacin produced extensive visible haemorrhagic necrosis of gastric mucosa; positive control group treated with misoprostol (0.1 mg/kg) (c) and Maalox (50mg/kg) (d): injuries to the gastric mucosa are very milder compared to the injuries seen in the ulcer control rats (arrow); tests groups treated with *A. pruinosum* (125 mg/kg) (e), treated with *A. pruinosum* (250mg/kg) (f), treated with *A. pruinosum* (500mg/kg) (g): extract reduces the formation of gastric lesions induced by indomethacin(arrow). Mild injuries to the gastric mucosa are seen, and flattening of the gastric mucosa is seen (arrow).

Indomethacin produced extensive visible haemorrhagic necrosis of gastric mucosa in ulcer control animals compared to normal animals treated with vehicle. Treatment with plant-extract or reference drugs decreased the severity of injuries seen in the gastric mucosa. In positive control group treated with Misoprostol (0.1 mg/kg) and maalox (50 mg/kg), injuries to the gastric mucosa were very milder compared to the injuries seen in the ulcer control rats. Extract reduces the formation of gastric lesions induced by indomethacin, mild injuries and flattening to the gastric mucosa were seen with different doses of *A. pruinosum* (Figure 1).

#### Effect of A. pruinosum on gastric lesions induced byindomethacin in rats

Oral administration of indomethacin caused marked mucosal lesions in rats’ glandular stomachs. Administration of *A. pruinosum* significantly (*p* < 0.05) reduced this mucosal lesion in rats’ stomachs. This reduction of ulcerations (mm^2^) was dose-dependent from 250 to 500 mg/kg b.w. Thus, ulcerations varied from 65.50 ± 6.05 mm^2^ (negative control group) to 28.00 ± 4.24 mm^2^ (125 mg/kg) and 16.00 ± 7.87 mm^2^ (500 mg/kg) of *A. pruinosum* (Table 5). *A. pruinosum* treatment significantly (*p* < 0.05) diminished the ulcer index of indomethacin-induced ulcerations relatively to negative control group. The mean ulcer index decreased from 4.88 ± 0.88 (negative control group) to 1.71 ± 0.44 (*A. pruinosum* at 500 mg/kg b.w.). *A. pruinosum* treatment also significantly (*p* < 0.05) diminished the percentage of ulcerated area of indomethacin-induced ulcerations relatively to the negative control group. The mean percentage of ulcerated area decreased from 8.15 ± 0.33% (negative control group) to 1.71 ± 0.44 (*A. pruinosum* at 500 mg/kg b.w.). Gastric lesions in rats’ stomachs were dose-dependent and significantly (*p* < 0.05) inhibited from 57.91% to 75.57% by treatment with *A. pruinosum* at doses varying from 125 to 500 mg/kg b.w. (Table 5), while Misoprostol (0.1 mg/kg b.w.) and Maalox (50 mg/kg b.w.) exhibited respective inhibition of 65.26% and 51.91%.

**Table 5.** Effect of *A. pruinosum* extract on gastric lesions induced by indomethacin in rats.

Treatment	UA (mm^2^)	UI	UA%	I%
Normal group	/	/	/	/
Negative control group	65.50 ± 6.05	4.88 ± 0.88	8.15 ± 0.33	/
Maalox (50 mg/kg)	31.50 ± 9.33*	2.88 ± 0.31*	6.78 ± 0.55*	51.91
Misoprostol (100μg/kg)	22.75 ± 5.80**	1.75 ± 0.26**	3.50 ± 0.09**	65.26
*A. pruinosum* (125 mg/kg)	28.00 ± 4.69*	3.06 ± 0.83*	6.04 ± 0. 39*	57.27
*A. pruinosum* (250 mg/kg)	21.75 ± 9.74**	2.25 ± 0.55*	4. 37 ± 0.25**	66.79
*A. pruinosum* (500 mg/kg)	16.00 ± 7.87***	1.71 ± 0.44**	1.14 ± 0.19***	75.57

The results are mean ± SD for 6 animals/group. Significantly different from the control group at *p < 0.05, **p < 0.01; ***p < 0.001. UI: Ulcer index; UA: total ulcer area; UA%: percentage of ulcerated area; I%: inhibition percentage.

## Discussion

In this study, the anti-ulcer properties of *A. pruinosum* on two most common aetiology of peptic ulcers, indomethacin as non-steroidal anti-inflammatory drugs and *H. pylori* as microbial antigens were evaluated.

The anti-*Helicobacter* activity of methanol and ethyl acetate extracts of the plant was performed using the broth microdilution assay. The anti-*Helicobacter* activity of the methanol and ethyl acetate extract of *A. pruinosum* showed MIC values ranging from 128 to >1024 μg/mL. The lowest MIC value of 128 μg/mL was recorded with the methanol extract of *A. pruinosum* against *H. pylori* α1 and *H. pylori* α2. The activity of the methanol extract demonstrated herein indicates that the anti-*Helicobacter* potential components of the tested plant are more soluble in methanol. Antimicrobials activity is considered to be significant if MIC values are below 100 μg/mL for crude extract and moderate when 100 < MIC <625 μg/mL (Kuete & Efferth 2010). In view of the above classification, the activity recorded with *A. pruinosum* methanol extract against *H. pylori* α1 and *H. pylori* α2 can be considered moderate. An alternative criterion has been described by Fabry et al. (1998), which consider extracts having MIC values below 8000 μg/mL to have noteworthy antimicrobial activity. Under these less stringent criteria, and considering the fact that the plant part tested are commonly eaten as ingredients for spirit protection with limited toxicity, the activity recorded could be considered as important. However, MIC values for the methanol extract of *A. pruinosum* against *H. pylori* α2 was less than those of ciprofloxacin, erythromycin, doxycycline and amoxicillin, standard drugs used. Phytochemical screening of methanol extract of *A. pruinosum* revealed the presence of phenols, tannins, triterpenes and anthraquinones (Table 2). These results are in accordance with previous results that indicated that major volatile components from seeds were oxygenated components belonging to the acyclic terpene class, such as (*E*)-nerolidol (91.7%) in the seeds oils (Amvam Zollo et al. 2005). The presence of these chemical compounds in the tested plant-extract may partially explain its anti-*Helicobacter* effect since it is documented that components with an alcohol functional group such as (*E*)-nerolidol and linalool were highly active against microorganisms (Brehm-Stecher & Johnson 2003; Schmidt et al. 2010). Indeed, tannins by inactivating microbial adhesions, enzymes, cell envelope transport protein exert their antimicrobial action while triterpenes and sterols increase permeability and loss of cellular components, including those involved in the production of cellular energy and synthesis of structural components (Linuma et al. 1993).

Chronic administration of non-steroidal anti-inflammatory drugs (NSAIDs) such as indomethacin, during the course of anti-inflammatory therapy, is often associated with the development of adverse gastrointestinal disorders such as gastric erosions, gastric or duodenal ulceration and other severe complications such as gastrointestinal haemorrhage or perforation that often limited their wide spread clinical use (Villegas et al. 2004). Indomethacin is known to induce gastric ulcer by inhibition of prostaglandins which are cytoprotective to gastric mucosa (Hoogerwerf & Pasricha 2001), particularly due to the inhibition of cyclooxygenase pathway of arachidonic acid metabolism resulting in excessive production of leukotrienes and other products of 5-lipoxygenase pathway (Villegas et al. 2004; Takeuchi 2014). In the stomach, prostaglandins play a vital protective role, stimulating the secretion of bicarbonate and mucus, maintaining mucosal blood flow, and regulating mucosal cell turnover and repair (Hiruma-Lima et al. 2006). Thus, the suppression of prostaglandins synthesis by indomethacin results in increased susceptibility to mucosal injury and gastric ulceration. Recently, reactive oxygen species (ROS) have also been shown to play a critical role in the development of pathogenesis in acute experimental gastric lesions induced by NSAIDs (Banarjee 1990).

In the present study, the significant increase in ulcer index and in percentage of ulcerated mucosal surface area following oral administration of indomethacin in the ulcerated rats may be attributed to either free radicals formation or inhibition of prostaglandin synthesis. This agrees with the reports of Bech et al. (2000), Biplab et al. (2011) and Muhammed et al. (2012) where indomethacin was reported to have caused alterations in gastric secretions of rats. Conversely, treatment with *A. pruinosum* methanol extract significantly reduced these parameters. In fact, the effects noticed for the mean percentage of ulcerated mucosal surface area, ulcer index and inhibition percentage compared favourably well with standard drug used in this study and indeed suggestive of their possible gastroprotective attributes.

A combination of events including release of preformed mucus, wound retraction and re-epithelialization is involved in ulcer-healing process after toxicological injury (Naito et al. 1995; Luo et al. 2013). The mucus offers protection against both endogenous aggressors and exogenous gastro-toxic agents such as indomethacin, thereby enhancing the rate of local healing process (Alanko et al. 1999). Thus, drugs that increase the synthesis and secretion of gastric mucus would accelerate gastric ulcer healing. Our data showed that, treatment with the tested plant facilitated ulcer healing process, which is associated with elevated gastric mucus level. This in turn encouraged speedy wound healing of the ulcerated areas of the mucosal epithelia and shielded the gastric membrane, thus abrogating the catastrophic influence of indomethacin in the ulcerated rats (Naito et al. 1995).

On the other hand, indomethacin-induced ulcer was associated with a decreased level of NO in rat’s gastric tissue in this study. Conversely, treatment with *A. pruinosum* methanol extract and reference drugs significantly replenished the depleted gastric NO level in such a way that its level was similar to that of normal animal, suggesting the possible mobilization and involvement of NO in the antiulcer effect of *A. pruinosum* since NO is known to play a role in maintaining gastric integrity by increasing mucosal blood ﬂow and mucus secretion (Bjorne et al. 2004), cytoprotection (Duranski et al. 2005; Shiva et al. 2007) as well as anti-inﬂammatory and antioxidant effects (Stokes et al. 2009; Carlstrom et al. 2011). Indeed, studies have shown that nitrite-rich saliva increases mucosal blood ﬂow and mucus generation in an NO-dependent manner (Bjorne et al. 2004; Petersson et al. 2007). Application of human nitrite-rich saliva or sodium nitrite to the rat gastric mucosa *in vivo* led to a dose-dependent increase in mucosal blood ﬂow and mucus generation which were both cGMP-dependent (Bjorne et al. 2004). Moreover, NO-promoting treatments have been shown to be beneficial in GI ischaemia-reperfusion injury models as well as in protection against upper GI bleeding induced by aspirin and non-steroidal anti-inﬂammatory drugs (NSAIDs) (Kurose et al. 1994; Panes & Granger 1998; Lanas et al. 2000). Taken together, these properties indicate important protective properties of NO and NOS-derived in the stomach. Thus, this plant-extract may also exert its antiulcerogenic effect by increasing endogenous NO generation in stomach through l-arginine-NO pathway and/or non-enzymatic protonation of swallowed salivary nitrite (Benjamin et al. 1994; Lundberg et al. 1994). However, the supposed nitrate content of the plant extract can boost gastric NO levels through the enterosalivary circulation of nitrate but, this needs to be further investigated. The overall activity recorded herein on *A. pruinosum* against indomethacin-induced ulcer is indicative of enhanced mucus secretory potential of *A. pruinosum,* and is suggestive of its significant role in ulcer healing process.

The phytoconstituents found in *A. pruinosum* were phenols, tannins, triterpenes and anthraquinones. These phytoconstituents present in *A. pruinosum* could be the possible agents in the treatment of ulcers induced in rats. Indeed, the phytoconstituents like triterpenoids and tannins are among the cytoprotective active substances for which antiulcerogenic efficacy has been extensively confirmed (Borellli & Izzo 2000; Nwagba et al. 2013). Tannins may prevent ulcer development due to their protein precipitating and vasoconstriction effects. Their astringent action can help precipitate micro proteins on the ulcer site, thereby forming an impervious layer and protecting the underlying mucosa from toxins and other irritants (Berenguer et al. 2005; Devaraj & Krishna 2011).

## Conclusions

The results of this study showed that methanol extract from the seeds of *A. pruinosum* possessed a moderate anti-*Helicobacter* and antiulcer activities against indomethacin-induced ulcer and could be a potential source for novel antiulcer drug discovery and development. The cytoprotective action of *A. pruinosum* may result from the strengthening of the mucosal barrier through the increase of the mucus production. The mechanism underlying this antiulcerogenic effect may be the stimulation of endogenous prostaglandins and/or NO generation in stomach.
